# The Intrinsic Innate Immunity of Hepatocytes Suppresses HBV Replication and Is Antagonized by HBx

**DOI:** 10.3390/v17121599

**Published:** 2025-12-10

**Authors:** Chui Zeng, Fayed Attia Koutb Megahed, Yiqiong Guo, Dongmei Sun, Yaru Wang, Qin Liu, Yanwei Bi, Jinghang Li, Qi Zhou, Qingdong Xie, Pingnan Sun, Xiaoling Zhou

**Affiliations:** 1 Stem Cell Research Center, Shantou University Medical College, Shantou 515041, China; zengchui@gwcmc.org (C.Z.); fkoutb@srtacity.sci.eg (F.A.K.M.); 21yqguo@stu.edu.cn (Y.G.); wyr03231026@163.com (Y.W.); 20qliu1@stu.edu.cn (Q.L.); 15ywbi@stu.edu.cn (Y.B.); li.jinghang.42w@st.kyoto-u.ac.jp (J.L.); 17qzhou3@stu.edu.cn (Q.Z.); qdxie@stu.edu.cn (Q.X.); 2 The Center for Reproductive Medicine, Shantou University Medical College, Shantou 515041, China; 3 Guangdong Provincial Key Laboratory of Infectious Diseases and Molecular Immunopathology, Shantou University Medical College, Shantou 515041, China; 4 Guangzhou Women and Children’s Medical Center, Guangzhou Medical University, Guangzhou 510120, China; 5 Department of Nucleic Acid Researches, Genetic Engineering and Biotechnology Research Institute, General Authority-City of Scientific Researches and Technological Applications, Alexandria 21934, Egypt; 6 Pingdi Public Health Service Center, Longgang District, Shenzhen 518117, China; 13686419440@163.com

**Keywords:** HBV, intrinsic innate immunity, HBx, *TRIM22*, *TRIM56*

## Abstract

(1) Background: Hepatitis B virus (HBV) belongs to the Hepadnaviridae family of viruses that interact with hepatocytes. HBV infection is a major global health problem. Most adults clear the infection quickly after being infected with HBV, while a few people develop chronic HBV infection. It is well-known that the early innate immune response of host cells plays an important role in the fight against virus infection. However, the interactions between HBV and the intrinsic innate immune system of hepatocytes are still not fully understood. The aim of this study was to confirm the interaction between HBV and hepatocytes, and to identify the interferon-stimulated genes (ISGs) regulated by HBx and their expression in association with HBV-associated HCC (HBV-HCC), so that we can refine our understanding of the interaction between HBV and ISGs and its potential influence on HBV-HCC. (2) Methods: We analyzed data concerning the stimulation of IFN-dependent genes in primary human hepatocytes (PHHs) transfected with pathogen DNA mimetics or infected with HBV in the GSE69590 database. Bioinformatic methods, such as GSEA, GO, and KEGG, were used to analyze the differentially expressed innate immunity genes and their related pathways to identify candidate intrinsic innate immune factors. qPCR on HepG2 and Huh7 cells, which highly express HBx, was used to detect relevant intrinsic innate immune factors. qPCR, RNAi, and Elisa methods were used to identify intrinsic innate immune factors in HBV-integrated HepG2.2.15 cells, and bioinformatics analysis was conducted on the HBV-infected tissues and cells in the GEO database. (3) Results: Inhibition of the JAK-STAT pathway enhanced HBV replication in HepG2 cells transfected with HBV plasmid and HepG2-NTCP cells infected with HBV. GSEA analysis of the GSE69590 data revealed significant changes in intrinsic innate immune pathways during HBV infection with PHH for 40 h. A total of 84 differentially expressed, candidate innate immunity genes were identified in GSE69590. Validation showed that *TRIM22* and *TRIM56* were down-regulated when HBx was expressed. Consistently, *TRIM22* and *TRIM56* were up-regulated following inhibition of HBx by transfection of HBx siRNA into HepG2.2.15 cells, and HBV pgRNA was up-regulated following down-regulated expression of *TRIM22* and *TRIM56* in HEK293 cells. Receiver operating characteristics (ROC) and overall survival (OS) analysis of 204 HBV-HCC patients showed that expression of *TRIM22* was closely associated with HBV-HCC, and high expression of *TRIM22* was associated with longer survival. (4) Conclusions: Innate immunity genes *TRIM22* and *TRIM56* are regulated by HBx, and higher expression of *TRIM22* is closely related to longer survival of HBV-HCC patients.

## 1. Introduction

Hepatocellular carcinoma (HCC) is one of the most prevalent malignancies in the world, accounting for 70–90% of primary liver cancers [1]. Approximately 54.4% of HCC cases are attributable to hepatitis B virus (HBV) infection [2]. Patients with chronic HBV have a high risk of hepatitis, cirrhosis, and hepatocellular carcinoma (HCC), the latter resulting in 692,000 deaths every year [3]. HBV is a member of the Hepadnaviridae family and is 3.2 kb in length. Its DNA is partially double-stranded and replicates its genome by reverse transcription. There are four overlapping open reading frames (ORFs) encoding seven proteins (pre-S1, pre-S2, S, pre-C, C, polymerase, and HBV x protein) [4]. HBV-associated liver damage is thought to be the consequence of a long-lasting cytolytic immune response against infected hepatocytes [5,6].

Interactions between HBV and the innate immune system of hepatocytes arise following HBV invasion. However, it is controversial whether early innate immune responses take part in the defense against HBV infection in hepatocytes. Several clinical and experimental studies have reported a lack of robust innate immune activation. For instance, no obvious increased concentrations of IFN-α and IFN-λ1 have been observed in serum of acute HBV-infected patients with high quantities of viral particles and antigens [7]. Cheng et al. did not observe an interferon (IFN) response in human cell-culture models and humanized Alb-uPA/SCID mice under conditions of robust HBV replication [8]. In contrast, other studies have documented innate immune recognition of HBV. Shlomai et al. detected the expression of type I and III IFNs as well as some ISGs involved in antiviral responses in primary human hepatocytes following HBV infection [9], While Yoneda et al. found that HBV infection elicited a rapid innate immune response characterized by the production of inflammatory chemokines in hepatocytes [10]. Furthermore, Sato et al. identified HBV pregenomic RNA as a specific inducer of type III interferon, with RIG-I acting as both a sensor and a direct suppressor of viral replication [11]. Thus, while evidence exists on both sides, the precise innate immune mechanisms that hepatocytes employ to counteract HBV are still not fully elucidated.

However, HBV is considered as a “stealth virus” that establishes persistent infection in hepatocytes by evading the host innate immune system [12]. HBV stimulates intrinsic innate immune responses by producing inflammatory chemokines in hepatocytes [10]. Pattern recognition receptors (PRRs) play an important role in initiating the innate immune response and activating the adaptive immune response [13]. PRRs in hepatocytes, such as cGAS-STING (the cyclic GMP-AMP synthase STING) and RIG-I-MAVS (mitochondrial antiviral signaling protein), can recognize and respond to DNA and RNA derived from invading HBV [11,14,15]. After monitoring HBV by the above sensors, an effective antiviral type I or III interferon response will be activated and exert a crucial role in the inhibition of HBV in hepatocytes. The JAK-STAT pathway is the common downstream signaling cascade for both type I and type III interferon responses [16]. Evidence suggests that viral infections such as HCV can be enhanced through modulation of the JAK-STAT pathway [17]. Therefore, we suppose that interferon responses to HBV in hepatocytes will be easily monitored by inhibiting JAK-STAT pathway-enhanced HBV infection, even if the response was weak.

In addition, a necessary condition for HBV survival in infected hepatocytes is evasion of recognition and clearance by cell-mediated innate immunity. HBV proteins, including HBV polymerase, X protein, core/precore, HBs and hepatitis B e antigen, antagonize innate immune factors of hepatocytes [18,19,20,21,22,23,24,25,26,27,28]. This is especially true for, X protein (HBx), a highly conserved protein consisting of 154 amino acids with a molecular weight of approximately 17 kDa, which is well known in regulating HBV replication, host cell fate, and influences the progression of hepatitis B. HBx is the first viral gene expressed after HBV enters the cell and is crucial for the establishment of HBV infection [29,30,31]. It may also play a critical role in helping HBV to escape from innate immune recognition by impairing intrinsic innate immune recognition [22,24,32,33].

In this study, we use bioinformatics to analyze the innate immune factors associated with HBV infection of PHH, and we select two enriched TRIM family members, *TRIM22* and *TRIM56*, for validation. We show that expression of HBx suppresses the expression of *TRIM22* and *TRIM56*, while HBx siRNA increases the expression of HBV pgRNA after suppressing *TRIM22* and *TRIM56.* We further show *TRIM22* and *TRIM56* are expressed in HBV-HCC patients and correlated with overall survival (OS). In this study, we identified the innate immune factors that interact with HBx.

## 2. Materials and Methods

Bioinformatics Analysis

Microarrays and immunogenes

The gene expression dataset GSE69590 [10] was downloaded from the GEO database (https://www.ncbi.nlm.nih.gov/geo/) (accessed on 1 June 2023) [34,35], which is based on the GPL570 platform ([HG-U133_Plus_2] Affymetrix Human Genome U133 Plus 2.0 Array). All samples dealt with PHH cells, each group had three samples, and each pair had an uninfected group and infected group. The samples were transfected with pathogen DNA mimetics (ISD/dsDNA90) for 12 h or infected with HBV for 40 h (MOI = 50). We used the GEO2R tool to identify genes differentially expressed at either 12 h of ISD/dsDNA90 DNA transfection or 40 h of HBV infection compared with untreated PHH control groups, respectively. The innate immunity gene dataset used for analysis was downloaded from InnateDB [36] (https://www.innatedb.com/).

GSEA, jvenn, GO, and KEGG analysis

GSEA analysis was performed using the WEB-based GEne SeT AnaLysis Toolkit (http://www.webgestalt.org/) for genes with an adjusted *p*-value (P_adj_) < 0.05 in the two sets of differential genes [37]. Venn diagrams were drawn via a free web-based platform (https://www.bioinformatics.com.cn) [38], and GO and KEGG analysis was performed on this platform for the differentially expressed genes identified [39,40]. The three datasets used for the analysis were deduplicated genes.

Overall survival and ROC analysis

GSE14520 survival data were downloaded from the GEO database, and survival data from HBV-related HCC clinical samples were selected to find the optimal survival cut-off value using X-tile 3.6.1 software [41]. Overall survival analysis and ROC analysis were performed using MedCalc Statistical Software version 20.100 (MedCalc Software Ltd., Ostend, Belgium; https://www.medcalc.org; 2022).

Cell culture and Plasmid

HepG2 and Huh7 human hepatocellular carcinoma cells (China Center for Type Culture Collection), HepG2-NTCP cells stably expressing Na^+^-taurocholate cotransporting polypeptide (NTCP) (HepG2-hNTCP), and HepG2.2.15 cells producing a higher level of HBV were cultured in Dulbecco’s modified Eagle’s medium (DMEM) (Thermo Fisher, Paisley, UK) containing 10% fetal bovine serum (FBS; Thermo Fisher), 100 IU/mL penicillin, and 100 μg/mL streptomycin (Merck, Millipore, Hertfordshire, UK) in a 5% CO_2_ incubator at 37 °C. The plasmid of pcDNA-HBV1.1 containing 1.1-copies of the HBV genotype C from the p3.6 II/HBV was a gift from Professor Wensheng Sun and Professor Xiaohong Liang [42] and was used in our previous study [43].

HepG2 transfection

HepG2 wells were cultured in a 12-well plate with appropriate medium. Medium was changed every 48 h until 60% confluence. Then, cells were co-transfected with p.max-EGFP (258.4 ng/mL and HBV1.1 (120 ng/mL) plasmids using a Lipofectamine 3000 transfection kit (Thermo Fisher Scientific, Waltham, MA, USA) according to the manufacturer’s instructions, and cells were incubated in a 5% CO_2_ incubator at 37 °C for 6–8 h, followed by refeeding with 1.5 mL fresh media. Media was then changed every 48 h. Supernatants were collected in 1.5 mL sterilized tubes for detection of HBV markers using ELISA and qRT-PCR. On day 10 post-transfection, the cells were fixed with ice methanol for total RNA extraction and immunofluorescence.

HepG2-NTCP infection with HBV particles

The HBV used in this study was of the genotype D strain and was derived from the culture supernatant of HepG2.2.15 cells. The culture supernatants of Hep2.2.15 cells were recovered and centrifuged for 15 min at 3500× *g* and cleared through a 0.45 μm filter to remove cell debris, followed by precipitation with 8% polyethylene glycol (PEG) 6000. The precipitates were washed and resuspended with the medium at approximately 100-fold concentration. The HBV DNA copy numbers were determined via real-time PCR analysis using a DAAN gene kit (DAAN GENE, Guang Zhou, China) according to the manufacturer’s instructions. HBV infection was performed as described below. Cells were inoculated with HBV at the indicated genome equivalent (GEq)/cell in the presence of 4% PEG8000 and 1% DMSO. Following a 24 h incubation period at 37 °C, the cells were gently washed 3 times with PBS and then cultured using the appropriate culture medium. Cells were washed and medium was changed every 2 days after infection. The culture medium was also replaced with fresh medium at 2, 4, 6, 8, and 10 days after infection. Supernatants were collected in 1.5 mL sterilized tubes for HBV marker detection by ELISA and qRT-PCR. On day 10 post-transfection, the cells were lysed with TRIzol™ reagent (Invitrogen, Carlsbad, CA, USA) for total RNA extraction, followed by quantification of HBV genetic markers and innate immune pathway genes using RT-qPCR.

Treatment of cells with JAKi

Cells (HepG2 and HepG2-NTCP) were treated with 5, 10, or 15 µg/mL polyinosine/polycytidylic acid (poly I:C, Tlrl-pic, InvivoGen, San Diego, CA, USA) and also treated with 5, 10, 15 µm/mL Janus kinase inhibitor 1 JAKi (MilliporeSigma, Burlington, MA, USA) during the transfection or infection of cells, and supernatants were collected on days 2, 4, 6 post-transfection or days 4, 6, 8, 10 post-infection.

Hepatitis B surface antigen (HBsAg) and hepatitis B e antigen (HBeAg) ELISAs

HBsAg and HBeAg levels in cell culture supernatants were quantified using CLIA ELISA Kits (Shanghai Kehua Bio-Engineering, Shanghai, China) according to the manufacturer’s protocol.

RNA extraction, quantification, RT-PCR, and qRT-PCR

Viral and cellular RNA from cells was extracted to determine gene expression or HBV replicative intermediate production and innate immunity genes using Viral RNeasy Plus Mini Kit or an RNeasy Plus Mini Kit (Qiagen, Hilden, Germany) according to the manufacturer’s protocol. RNA concentration and purity were determined using a NanoDrop 2000 spectrophotometer (Thermo Fisher Scientific, Wilmington, DE, USA). cDNA was synthesized from 1000 ng of total RNA using ReverTra Ace qPCR RT kit (TOYOBO, Osaka, Japan) as described by the manufacturer’s protocol. qPCR quantification of HBV pgRNA and HBV mRNA, HBx, HBVp genes, and different innate immunity pathway genes were performed using PowerUp SYBR Green Master Mix (Applied Biosystems, Thermo Fisher Scientific, Waltham, MA, USA) and using a Real-Time PCR System instrument (Applied Biosystems, USA) following the manufacturer’s protocol. Gene expression levels were determined using TaqMan Real-Time PCR assays (Life Technologies, Thermo Fisher Scientific, Waltham, MA, USA). The level of expression in each case was normalized to the housekeeping gene GAPDH and read as CT to calculate 2^−ddCT^ as the level of gene expression. Primer sequences are shown in Supplemental Table S1.

siRNA down-regulation of *TRIM22* or *TRIM56* in HEK293 cells

Lentiviruses were prepared in HEK293 cells and concentrated by ultracentrifugation, followed by use in HepG2 and Huh7 hepatoma cell transduction. After lentiviral transduction, cell were treated with 50 ng/mL IFN-λ1, and the effect of HBx on type III interferon response in hepatoma cell lines was analyzed via qPCR. The siHBx interfering RNA was synthesized and transfected into HepG2.2.15 cells. The supernatant was collected, and an ELISA was used to detect changes in HBsAg and HBeAg concentrations in the cell supernatant. RNA was extracted and qPCR was used to detect HBx, *TRIM22*, and *TRIM56*. Chemical synthesis of ISG siRNAs was performed and transfected into HEK293 cells, followed by transfection of HBV plasmid and addition of IFN-λ1. RNA was collected and qPCR was used to detect pgRNA. siRNA sequences are shown in Supplemental Table S1.

siRNA down-regulation of HBx in HepG2.2.15 cells

Twelve hours after cell inoculation, the cells were replaced with Opti-MEM I medium and then transfected. RNA was collected for qPCR 24 h after transfection. ELISAs were performed 48 h after transfection. The siRNA doses were 50 and 100 nM. siRNA sequences are shown in Supplemental Table S1.

Statistical analysis

All experiments were carried out in at least three replicates. Data are expressed as the mean ± standard deviation (SD). A Microsoft Excel database and SPSS 20.0 software were used to record and analyze all data. One-way ANOVA and Student’s *t*-test were used for data analysis. Differences with a *p*-value of <0.05 were considered statistically significant.

## 3. Results

### 3.1. Modulation of Intrinsic Innate Immunity Increases HBV Replication in HepG2 and HepG2-NTCP

**Figure 1:**
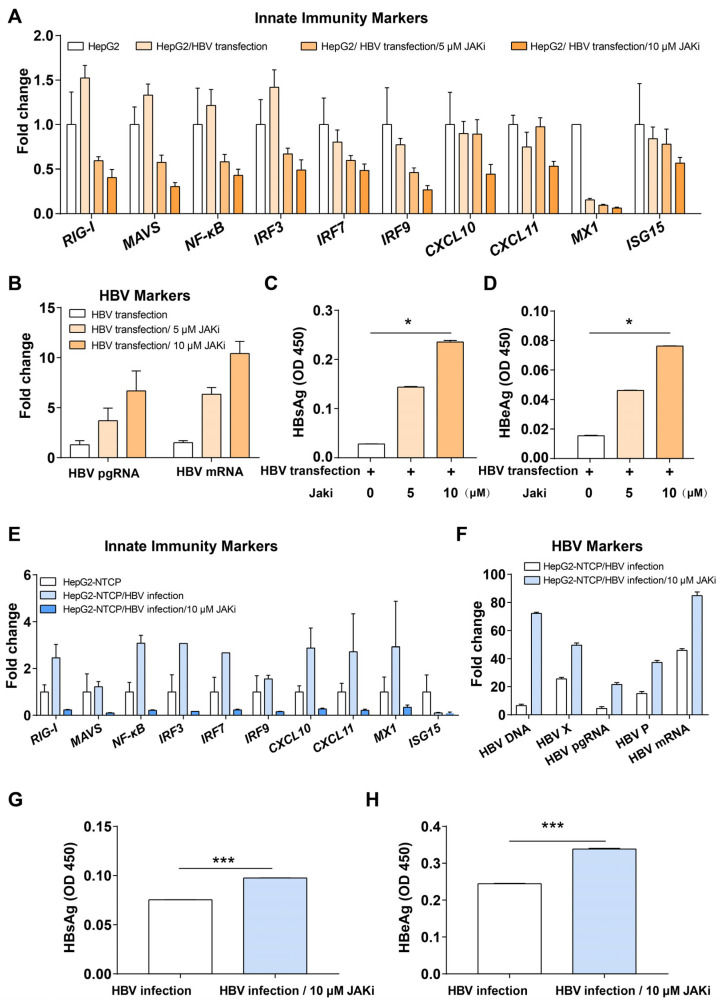
Inhibition of JAK-STAT pathway improved HBV replication. (**A**) Innate immunity markers in HepG2 cells, HepG2 cells transfected with HBV, HepG2 cells transfected with HBV and treated with JAKi (5 or 10 μM). (**B**) Expression of HBV markers in JAKi-treated or non-treated HepG2 cells transfected with HBV. (**C**) ELISA for HBsAg in JAKi-treated or untreated HepG2 cells transfected with HBV for 6 days. (**D**) ELISA for HBeAg in JAKi-treated or untreated HepG2 cells transfected with HBV for 6 days. (**E**) Innate immunity markers in HepG2-NTCP cells, HepG2-NTCP cells infected with HBV, and HepG2-NTCP cells infected with HBV and treated with JAKi (10 μM). (**F**) Expression of HBV markers in treated or untreated HepG2-NTCP cells infected with HBV. (**G**) ELISA for HBsAg in JAKi treated or untreated HepG2-NTCP cells infected with HBV for 10 days. (**H**) ELISA for HBeAg in JAKi-treated or untreated HepG2-NTCP cells infected with HBV for 10 days. * represents *p* < 0.05, *** represents *p* < 0.001.

In the above bioinformatics analysis, we found that intrinsic innate immune pathways, including type I interferon-related pathways, were activated during HBV infection of PHH. The JAK-STAT pathway is downstream of both type I and III interferon pathways, which is the major interferon response of PHH. Therefore, we treated both HepG2 cells transfected with HBV plasmid and HepG2-NTCP cells infected with HBV with JAK inhibitor I (JAKi, an inhibitor of JAK-STAT pathway) to modulate the interferon pathway and examine whether it would promote HBV gene expression. The results showed that the expression of innate immune genes, such as *RIG-I*, *MAVS*, *NF-κB*, *IRF3*, *IRF7*, and *IRF9*, were down-regulated in JAKi-treated cells compared with the control group (Figure 1A,E), while the expression of HBV pgRNA and HBV mRNA was increased (Figure 1B,F). The expression of secreted HBsAg and HBeAg was also increased (Figure 1C,D,G,H).

### 3.2. Innate Immunity Genes Involved in HBV Infection

**Figure 2:**
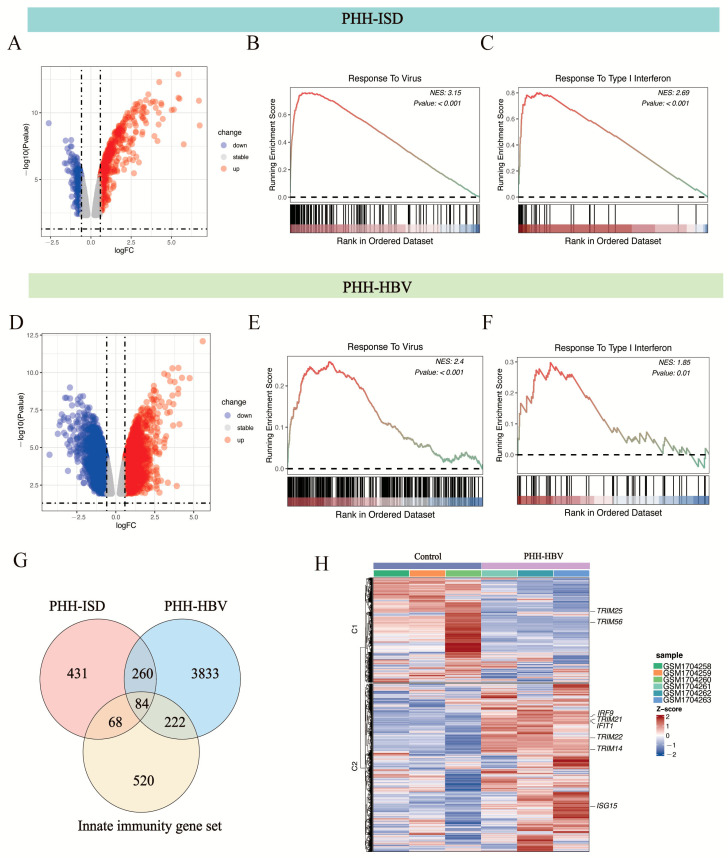
GSEA analysis of differentially expressed genes (Padj < 0.05) in ISD-transfected and HBV-infected PHH (GEO accession number: GSE69590 [10]). (**A**) Volcano plot showing the up-regulated (red spot) and down-regulated (blue spot) proteins between PHH-ISD/dsDNA90 (12 h) cells and PHH cells. Differentially expressed genes that showed a 1.5-fold or greater change and *p*-value less than 0.05 were considered significantly up- or down-regulated. (**B**) Response to virus biological process was significantly enriched in PHH-ISD/dsDNA90 (12 h) cells. (**C**) Response to type I interferon biological process was significantly enriched in PHH-ISD/dsDNA90 (12 h) cells. (**D**) Volcano plot showing the up-regulated (red spot) and down-regulated (blue spot) genes between PHH-HBV (40 h) cells and PHH cells. Differentially expressed genes that showed a 1.5-fold or significantly up- or down-regulated. (**E**) Responses to virus biological change with *p*-values less than 0.05 were considered processes significantly enriched in PHH-HBV (40 h) cells. (**F**) Responses to type I interferon biological processes were significantly enriched in PHH-HBV (40 h) cells. (**G**) Identification and enrichment analysis of the 84 HBV-dependent, differentially expressed innate immunity genes common to ISD-transfected and HBV-infected PHH. (**H**) Heatmap of the expression of 84 overlapping genes. The normalized enrichment score (NES) is color-coded in (**B**,**C**,**E**,**F**); red represents high positive enrichment, and blue represents low or negative enrichment.

The GEO2R tool was used to analyze two HBV-infected/non-infected paired groups in the GSE69590 database [10], which contained data for PHHs transfected with pathogen DNA mimetics ISD/dsDNA90 for 12 h and PHHs infected with HBV (MOI = 50) for 40 h, and yielded 2570 and 3815 differential genes that had significant changes (P_adj_ < 0.05). We further performed GSEA analysis of these two groups of differentially expressed genes using WebGestalt [37] and found that both groups were enriched in response to virus and type I interferon biological processes (Figure 2A–F).

We further overlapped the statistically significant differentially expressed genes between PHH-ISD vs. PHH cells, the differentially expressed genes between PHH-HBV vs. PHH cells, and the innate immunity gene set. The overlap contained 84 innate immunity genes (Figure 2G). GO analysis of the 84 innate immunity genes showed that these genes were enriched in response to virus and type I interferon biological processes, such as *DDX58 (RIG -I)*, *IRF9*, *TRIM22*, *OAS1*, and *MX1* (Figure 2G,H). Notably, several tripartite motif (TRIM) family members such as *TRIM22*, *TRIM25*, *TRIM56,* and *TRIM14* are enriched during the process (Figure 2G,H). The TRIM family of proteins, which contain conserved RING finger, B-box, and coiled-coil structural domains, are involved in a variety of cellular processes, including cell proliferation, differentiation, development, tumorigenesis, apoptosis, and antiviral defense [44,45,46].

### 3.3. HBx Interrupts the Induction of ISGs in IFN-λ1-Treated Hepatocytes

**Figure 3:**
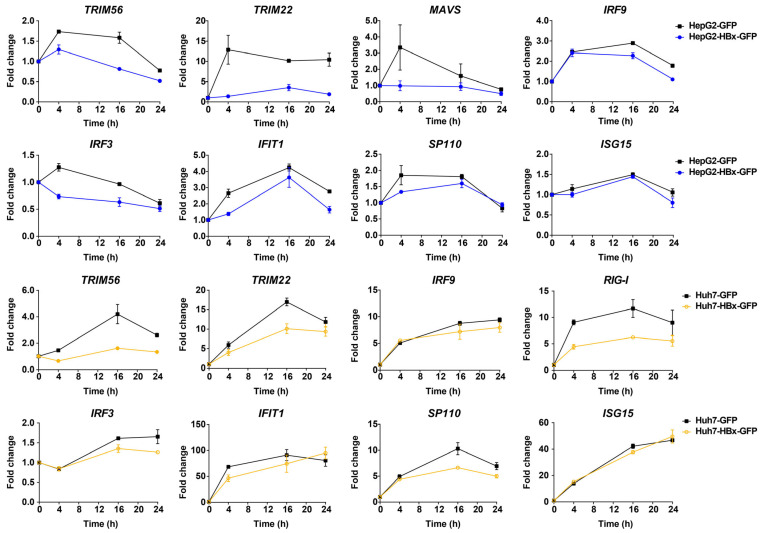
HBx-down-regulated genes related to the type III interferon response signaling pathway in HepG2 and Huh7 cells. RNA was respectively collected and extracted at 0, 4, 16, and 24 h after adding 50 ng/mL IFN-λ1. After reverse transcription, changes in ISGs were detected using qPCR, and the kinetics of changes in ISGs induced by IFN-λ1 were plotted using the respective 0 h as control.

From the above analysis, we know that innate immune signaling pathways play an important role in suppressing HBV when it invades the host’s cells. HBx may play a key role in HBV replication, helping HBV escape recognition by the intrinsic innate immune system and promoting HCC development. To investigate the interaction between HBx and innate immunity genes, we added IFN-λ1 to HepG2 and Huh7 cells, constitutively expressing HBx, to trigger interferon responses in hepatocytes, then we detected the effect of HBx on the expression of ISGs after 0, 4, 16, and 24 h. In HepG2 cells, HBx was able to reduce the induction of some interferon-related pathway genes, including *MAVS*, *IRF9*, *IRF3*, *IFIT1*, *TRIM56*, *TRIM22*, and *SP110*, by IFN-λ1 (Figure 3). Similarly, in Huh7 cells, HBx reduced the expression of IFN-λ1 interferon-related pathway genes *RIG-I*, *IFIT1*, *IRF3*, *IRF9*, *TRIM56*, *TRIM22*, and *SP110* (Figure 3).

### 3.4. Suppression of HBx Increases TRIM22/TRIM56 in HepG2.2.15

**Figure 4:**
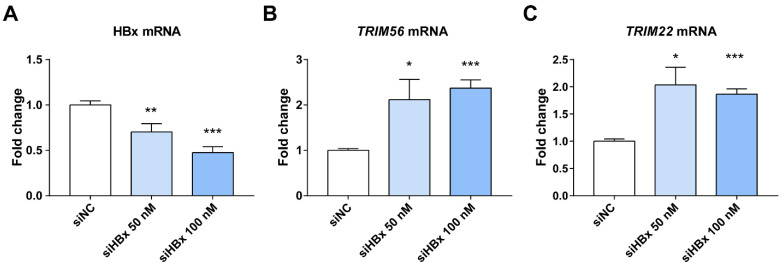
Effects of HBx knockdown on HBx and ISGs in HepG2.2.15 cells. (**A**) HBx was successfully knocked down, shown using qPCR, after transfection with 50 and 100 nM siHBx for 48 h. (**B**,**C**) Knockdown of HBx in HepG2.2.15 increased the expression of TRIM22 and TRIM56. * represents *p* < 0.05, ** represents *p* < 0.01, *** represents *p* < 0.001, *n* = 4, independent sample *t*-test.

We found above that HBx inhibits the expression of both *TRIM22* and *TRIM56* ISGs. Both of them have a RING finger E3 ubiquitin ligase [47,48]. HBx has also been reported in the literature to help HBV replication and transcription by inhibiting the transcription of *TRIM22* by methylating a single CpG at its 5′ end [23]. *TRIM56* is closely related to the cGAS-STING pathway as well as the TLR3 antiviral signaling pathway [49,50,51]. *TRIM56* plays a key role in recognition of dsDNA and dsRNA, and it is able to influence the expression of other ISGs. Therefore, we selected *TRIM22* and *TRIM56* to verify that HBx enhances HBV replication and expression by down-regulating *TRIM22* and *TRIM56*. qPCR results showed that knocking down HBx in HepG2.2.15 increased the expression of *TRIM22* and *TRIM56*, suggesting HBx inhibits the expression of *TRIM22* and *TRIM56* (Figure 4). In contrast, the expression of HBsAg and HBeAg secreted by HepG2.2.15 decreased after HBx knockdown (Supplemental Figure S1).

### 3.5. Down-Regulation of TRIM22 or TRIM56 Increases the Expression of HBV pgRNA

**Figure 5:**
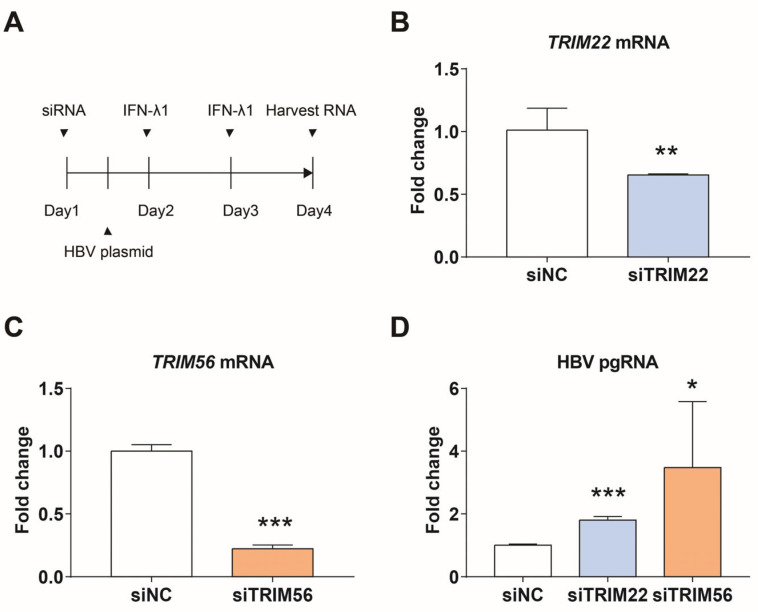
*TRIM22* and *TRIM56* regulate the expression of HBV pgRNA. (**A**) Scheme for siRNA knockdown and induction of interferon response in HEK293 cells. (**B**) Efficiency of siTRIM22. (**C**) Efficiency of siTRIM56. (**D**) Expression of HBV pgRNA after knocking down *TRIM22* or *TRIM56*. * represents *p* < 0.05, ** represents *p* < 0.01, *** represents *p* < 0.001, *n* = 4, independent sample *t*-test.

We tried to study the effect of knocking down *TRIM22* or *TRIM56* on HBV replication in HepG2.2.15 cells, but the results were not clear. The expression of *TRIM56* was found to be lower in HepG2.2.15 cells than in immortalized non-cancer HEK293 cells, which have a more intact intrinsic innate immune system. *TRIM56* was also lower in other hepatocellular carcinoma cell lines, such as HepG2 and Huh7 cells, than in HEK293. *TRIM22* is expressed lower in Huh7 than in HEK293 cells. Therefore, we next knocked down *TRIM22* or *TRIM56* in HEK293 cells, transfected with HBV plasmid, and added IFN-λ1 for 24 h after transfection. The relative levels of *TRIM22*, *TRIM56*, and HBV pgRNA were measured using qPCR 72 h after transfection of si*TRIM22* or si*TRIM56*. As shown in Figure 5, *TRIM22* and *TRIM56* were knocked down by approximately 35% and 78%, respectively, resulting in 1.7- and 3.4-fold increases in HBV pgRNA levels compared to the control.

### 3.6. Expression of TRIM22 and TRIM56 in the Tissues and Cells of HBV-Related HCC Patients

We further analyzed the changes in *TRIM22* and *TRIM56* expression in HBV-infected hepatocytes and HCC tissues, and we found that *TRIM22* and *TRIM56* expression was down-regulated in some HBV-infected hepatocytes and HCC tissues in the public database (Table 1).

**Figure 6:**
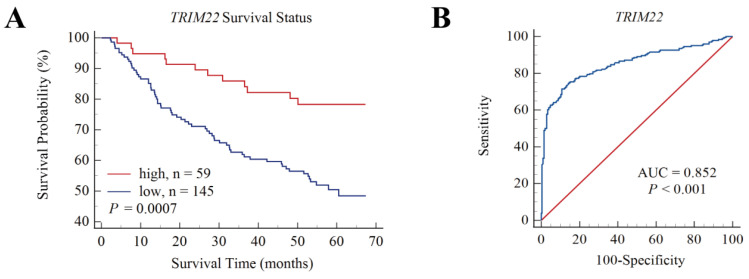
Overall survival (OS) and ROC of TRIM22 for HBV-associated HCC patients in GSE14520. (**A**). OS of TRIM22 shows survival rate of the high-TRIM22-expression group was higher than the low-TRIM22-expression group. (**B**). ROC of TRIM22, AUC = 0.852, *p* < 0.001.

Survival data were analyzed for *TRIM22* and *TRIM56* using HBV-associated HCC samples in GSE14520. The optimal survival cut-off value of 7.408 for *TRIM22* was determined using X-tile software, and the samples were divided into high- and low-expression groups. The survival graphs were further calibrated using MedCalc. Figure 6 shows that the OS in the high-expression group was higher than in the low-expression group (*p* = 0.0007), and receiver operating characteristic (ROC) analysis showed an area under the operating characteristics (AUC= 0.852, *p* < 0.001), indicating that *TRIM22* is highly expressed in HBV-associated HCC when patients had a high survival rate. In contrast, the association between high and low expression with OS of *TRIM56* was not significant (*p* = 0.1346) and did not distinguish well between HCC and non-HCC (AUC = 0.627, *p* < 0.001)).

## 4. Discussion

**Figure 7:**
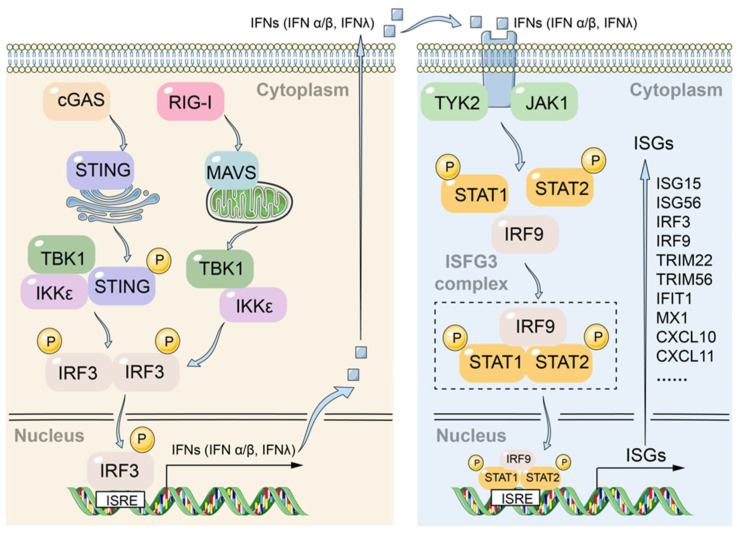
Crosstalk between the DNA-sensing cGAS-STING and RNA-sensing RIG-I/MAVS pathways converges on JAK-STAT signaling to orchestrate antiviral responses.

Innate immune responses triggered by the cellular intrinsic system such as PRRs that identify pathogen infection is essential for early clearance of hepatitis B virus infection. The cGAS-STING pathway is responsible for sensing viral DNA, while the RIG-I/MAVS pathway detects viral RNA. Upon activation, both pathways converge to phosphorylate the transcription factor IRF3 through the TBK1/IKKε kinases, thereby initiating the transcription and secretion of type I/III interferons. These interferons subsequently activate the JAK-STAT pathway, leading to the transcription of interferon-stimulated genes (ISGs) (Figure 7). In response to the host cell response, HBV has developed various strategies to evade the innate immune system to establish a chronic infection state in hepatocytes. Therefore, enhancing the intrinsic innate immune response of host cells may be an effective strategy for the development of new anti-HBV drugs, and several innate immunity activators such as Toll-like receptor and RIG-I agonist are being tested in clinical trials [56,57].

During the early stages of infection, interferon (IFN) is the main effector cytokine against various pathogens during the innate immune response. All types of IFNs (type I: IFN-α, IFN-β, and IFN-ω; type II: IFN-γ; type III: IFN-λ1 (IL-29), IFN-λ2 (IL-28A), and IFN-λ3 (IL-28B)) are induced mainly by pathogen-associated molecules activated by PRRs and share downstream signaling pathways, with receptors for type III interferons being hepatocyte enriched [58]. IFNs secreted by hepatocytes in response to viral infection eventually induce the expression of hundreds of ISGs that exert antiviral effects. Thus, ISGs often become viral protein targets, and inhibition of ISGs with anti-HBV effects aids viral evasion of hepatocyte intrinsic innate immunity. However, unknown anti-HBV intrinsic innate immune factors in hepatocytes have not been fully identified.

Our results suggest that when HBV invades hepatocytes, intrinsic innate immune factors of hepatocytes play a role in suppressing HBV. When we applied JAKi to inhibit the JAK-STAT pathway, we found that replication of HBV is enhanced (Figure 1). After we performed GSEA analysis of HBV-infected PHHs in GSE69590, we found that HBV infection causes an interferon response in PHHs (Figure 2). Previously, we used a Jak inhibitor to regulate the JAK-STAT pathway in hESC-derived hepatocytes, resulting in increased HCV infection in hepatocytes [17]. Recently, Carpentier A et al. also built a HCV infection model using a Jak inhibitor [59]. All these findings confirm that the intrinsic innate immunity system, the first defense line of hepatocytes, plays an important role in anti-HBV or HCV invasion.

In contrast, despite the fact that HBV DNA is a substrate for the cGAS-STING pathway, many studies have shown that HBV infection itself does not induce a robust innate immune response in human livers [60] or in infected primary human hepatocytes (PHH) [8,61,62,63]. Some studies have failed to detect innate immune responses in HBV-infected host cells, while others have observed such responses [10,11,64,65]. These divergent findings may be influenced by various experimental conditions—such as the stage of infection (acute or chronic), HBV genotype, viral load, and the source of host cells. Therefore, it is possible that HBV genuinely does not trigger innate immunity in host cells, or it may be that the virus has evolved strategies to evade or counteract the surveillance and effector functions of the innate immune system.

For example, HBx has been shown to suppress p53 expression in hepatocytes [66,67] and stabilize Nrf2 [68]. This mechanism may instead help hepatocytes counteract oxidative stress and associated innate immune responses. In our recent study, we found that HBx induces DNA double-strand breaks and triggers oxidative stress in embryonic stem cells by interacting with the DNA repair factor DDB1, leading to extensive apoptosis [33]. However, we did not observe this phenomenon in HBx-expressing hepatocytes, suggesting that hepatocytes possess unique regulatory mechanisms to manage inflammation or innate immune responses triggered by HBx.

Furthermore, our research demonstrated that treatment with the JAK inhibitor, which blocks innate immune signaling, resulted in increased HBV replication and gene expression. This provides evidence that the intrinsic innate immune system in hepatocytes does play a role in suppressing HBV.

HBx is considered to be the earliest expressed viral gene of HBV, critical for the establishment of HBV infection in hepatocytes [30,31]. We then targeted HBx, a key HBV protein that affects HBV replication and HCC progression, to uncover how HBx regulates the intrinsic innate immune system of hepatocytes. We constructed HepG2 and Huh7 hepatocellular carcinoma cells that stably overexpressed HBx and investigated the effect of HBx on the type III interferon response in both cells. When we overexpressed HBx, HBx down-regulated the stimulatory effect of type III interferon IFN-λ1 (IL-29) on *IFIT1*, *MAVs*, *RIG-1*, *IRF9*, *IRF3*, *TRIM22*, *TRIM56*, *SP110* in HepG2 cells, as well as down-regulated *IFIT1*, *RIG-1*, *IRF9*, *IRF7*, *IRF3*, *MX2*, *TRIM22*, *TRIM56*, and *SP110* in Huh7 cells (Figure 3). These intrinsic innate immune genes are involved in the following RIG-1/MDA5 pathway: *RIG-1*, *MAVs*, and *IFIT1*; and general interferon-related signaling pathways: *IRF3*, *IRF7*, *IRF9*, *OAS*, *MX2*, *TRIM22*, and *TRIM56*.

Previous studies have shown that HBx can help HBV escape the monitoring and attack of intrinsic innate immune system through different strategies, and it plays an extremely important role in the process of HBV becoming a “stealth virus” [69]. HBx can disrupt RIG-I-mediated IFN induction by down-regulating MAVS or attenuating the interaction between RIG-I and TNF receptor associated factor 3 (TRAF3) [21,22,70]. HBx can reduce TLR3 (Toll-like receptor-3) adaptor TRIF (TIR-domain-containing adaptor inducing interferon-beta) expression in a dose- and proteasome-dependent manner, thereby attenuating the TLR3 signaling pathway to promote HBV replication and allow the virus to evade the innate immune system [24]. MAVS is another PRR that recruits the kinases required for IFN production. HBx binds to MAVS and reduces IFN signaling. This is another possible means by which HBV can evade the immune system [22]. Recently, HBx was shown to transactivate ADAR1 via the transcription factor YY1. ADAR1 then binds and edits HBV RNAs, thereby blocking their recognition by RIG-I/MDA5 and ultimately suppressing the IFN signaling pathway [71]. The HBV1.1 strain used in our study belongs to Genotype C, which is prevalent in Asia. While HBx is one of the most conserved proteins of HBV, and its core functions remain consistent, amino acid sequence variations do exist across genotypes. Indeed, sequence differences in HBx among genotypes (e.g., A, C, D) may contribute to their distinct interferon treatment outcomes. In future studies, it would be valuable to compare how HBx proteins from different HBV genotypes vary in their regulation of innate immune responses.

Notably, we found enrichment of TRIM family members through bioinformatics analysis of 84 innate immune genes that significantly changed expression in HBV-infected hepatocytes. TRIM family proteins are induced in response to IFNs and are involved in a variety of biological processes, including tumorigenesis, apoptosis, and antiviral immune response [44,72]. Therefore, we selected two ISGs downstream of the interferon pathway, *TRIM22* and *TRIM56*, as targets for further study to examine their effects on HBV replication. *TRIM22* was associated with the clearance of HBV in chimpanzees infected with HBV [73]. *TRIM56* plays an important role in the cyclic GMP-AMP synthase STING (cGAS-STING) signaling pathway through which hepatocytes can innately immune respond to HBV and inhibit HBV assembly [15,49,50].

Our results showed that *TRIM22* was strongly induced by IFN-λ1. Compared with the control group, the expression of HBx in HepG2 and Huh7 decreased the expression levels of *TRIM22* and *TRIM56*. We transfected HBV plasmid into HepG2 cells with or without JAKi and examined the expression of *TRIM22* and *TRIM56*. Both the expression of TRIM22 and TRIM56 decreased in the HBV and JAKi treatment group compared with the HBV group (Supplemental Figure S2). When we knocked down HBx in HepG2.2.15 cells, release of HBsAg and HBeAg into the supernatant decreased, indicating that HBx is important for HBV replication in HepG2.2.15 cells. The levels of *TRIM22* and *TRIM56* in HepG2.2.15 cells were increased after knockdown of HBx, indicating that HBx has an inhibitory effect on *TRIM22* and *TRIM56* (Figure 4). We then knocked down *TRIM22* and *TRIM56* in HEK293 and HepG2 cell lines, respectively. qPCR results showed that knocking down *TRIM22* or *TRIM56* increases the expression of HBV pgRNA (Figure 5). We found that IL-29 (IFN-λ1) induced the expression of *TRIM22* and *TRIM56*, and HBV suppressed both of them in HEK293 cells 24 h after IL-29 treatment (Supplemental Figure S3). We further validated *TRIM22* and *TRIM56* down-regulation in HCC tissues or hepatocytes compared to paracancerous tissues or cells in other public databases. In conclusion, HBx helps HBV escape from the innate immune response by down-regulating *TRIM22* or *TRIM56*.

The area under the operating characteristics of the HBV-related HCC patients in GSE14520 was AUC = 0.852, *p* < 0.001, which could distinguish HCC cancer from non-cancer. Consistently, the expression of *TRIM22* in liver tissue and serum of 152 Korea HCC patients mostly with HBV infection is significantly lower than that of control patients with liver cirrhosis. The AUC of *TRIM22* diagnosis of liver cancer is 0.924, which can distinguish liver cirrhosis patients with normal HCC and normal AFP [74]. Therefore, the expression level of *TRIM22* is closely related to the development of HBV-related HCC and the survival of patients.

Actually, HBV and TRIM22 interacted with each other. HBV can repress *TRIM22* transcription through HBx-dependent methylation of a single CpG in the 5′-UTR of *TRIM22* mRNA [23]. TRIM22 inhibits the activity of four hepatitis B virus gene promoters, especially the S and C promoters, through the RING domain, thus effectively reducing the expression of HBsAg and HBeAg [75].

Hepatocytes mount an innate immune response to HBV that blocks viral assembly via the cyclic GMP-AMP synthase STING (cGAS-STING) signaling pathway [15].

*TRIM56 E3* mediates monoubiquitination of cGAS and enhances the induction of multiple ISGs downstream of IFN, further helping host cells to establish an antiviral state [50,76]. Overexpression of *TRIM56* enhances activation of the IFN-β promoter after dsDNA stimulation, and knockdown of *TRIM56* eliminates this effect [49]. HBV polymerase suppresses DNA-sensing pathways by directly interacting with STING and destroying the ubiquitin of its K63 connection [77]. Recently, Tian et al. found that TRIM56 impaired HBV infection and replication in HepG2 and PHH. Exogenous expression of *TRIM56* in HepG2 cells reduced the promoter activity of HBV PreS2, HBc, and HBx. When knocking down *TRIM56*, the promoter activity of PreS1, PreS2, HBc, and HBx increased [78]. However, Zhang et al. did not find that exogenous expression of *TRIM22* and *TRIM56* had effects on HBV replication when screened and identified TRIM family members with anti-HBV function in HepG2 [79]. The difference between these results may be caused by the expression level of *TRIM22* and *TRIM56* or the replication level of HBV in host cells. When analyzing the interferon response to IFN-α/β in PHHs, we found that the expression level of *TRIM22* changed much more than that of *TRIM56* after interferon induction (Table 2). In other words, *TRIM22* is a gene strongly induced by interferon, but *TRIM56* is not. This may also be one of the reasons why *TRIM56* has no significant influence on the overall survival of patients with HBV-related HCC.

In addition, the defects in the innate immune system in hepatoma cell lines from different labs may also result in the different results. Therefore, hepatocytes derived from stem cells or PHH with an intact innate immune system will be good alternative cell models in future studies [82]. They will facilitate the discovery of new innate immune factors against HBV, and they will provide further confirmation of the anti-HBV effects of *TRIM22* and *TRIM56*. Future research utilizing co-culture systems or animal models will be essential to elucidate how the cell-intrinsic immune suppression mechanisms in hepatocytes and intercellular immune activation signals collectively determine the outcome of HBV infection.

Based on the above analysis, *TRIM56* and *TRIM22* may contribute to the establishment of an anti-HBV state in the innate immune system. Additionally, the expression of *TRIM22* in hepatocytes can distinguish HCC from non-HCC, and the survival of HCC patients with high expression for *TRIM22* is longer than that of those with low expression. This indicates that the expression of intrinsic innate immune factors may help to resist the progression of HCC.

This study deepens and improves our understanding of the interactions between HBx and intrinsic innate immune factors of hepatocytes, providing a basis for future screening of HBx-interacting intrinsic innate immune factors, which may be helpful in predicting the progress of HBV-related HCC or developing new treatment strategies.

## Figures and Tables

**Table 1:** logFC values of *TRIM22* and *TRIM56* in other HBV-related databases and P_adj_.

	GSE14520 [52] Tissue	GSE84402 [53] Tissue	GSE121248 [54] Tissue	GSE55092 [55] Tissue	GSE55092 [55] Cell	Padj
*TRIM22*(logFC)	−1.81	−1.51	−0.94	−1.57	−1.57	<0.05
*TRIM56*(logFC)	0.38					<0.05

**Table 2:** IFN-α/β induced the expression of *TRIM22* and *TRIM56* in PHHs.

	GSE180646 [80]	GSE163042	GSE126090 [81]	GSE52752
*TRIM22*(logFC)	2.7284365	2.0612795	3.26	2.3469416
Padj.(TRIM22)	1.16 × 10^−17^	0.0155509	2.20 × 10^−11^	2.50 × 10^−6^
*TRIM56*(logFC)	0.2710238	0.69630274	0.949	0.9618422
Padj.(TRIM56)	0.0403	0.4714832	8.00E-07	0.00102

Padj. < 0.05 is considered significant.

## Data Availability

The original contributions presented in this study are included in the article. Further inquiries can be directed to the corresponding authors.

## Supplementary Materials

The following supporting information can be downloaded at: https://www.mdpi.com/article/10.3390/v17121599/s1, Figure S1: siHBX decrease HBsAG and HBeAg; Figure S2: TRIM22 and TRIM56 expression upon treatment with JAKi (10 μM) and HBV transfection for 48 h in HepG2; Figure S3: IFN-λ1 treatment induces TRIM22 and TRIM56 expressions in HEK293 cells with or without HBV transfection; Table S1: Primers or siRNA sequences.
