# Supplementary material for: The Intrinsic Innate Immunity of Hepatocytes Suppresses HBV Replication and Is Antagonized by HBx
Source: Viruses. 2025 Dec 10;17(12):1599. doi: 10.3390/v17121599 (PMC12737693; doi:10.3390/v17121599)
Supplement: Supplementary file 1 [file viruses-17-01599-s001.zip › viruses-3990587-supplementary.pdf]

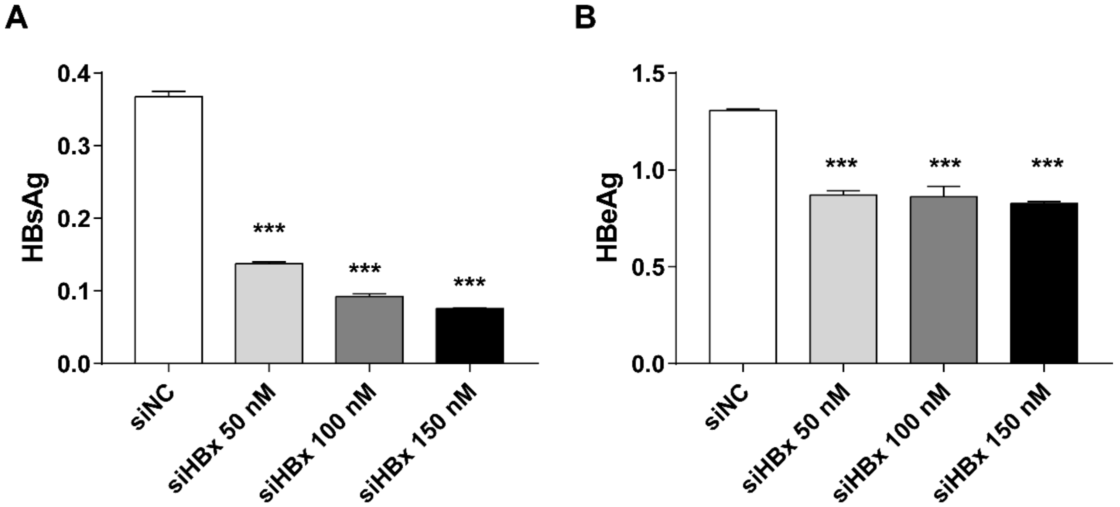

Supplemental Figure S1. siHBX decrease HBsAG and HBeAg.

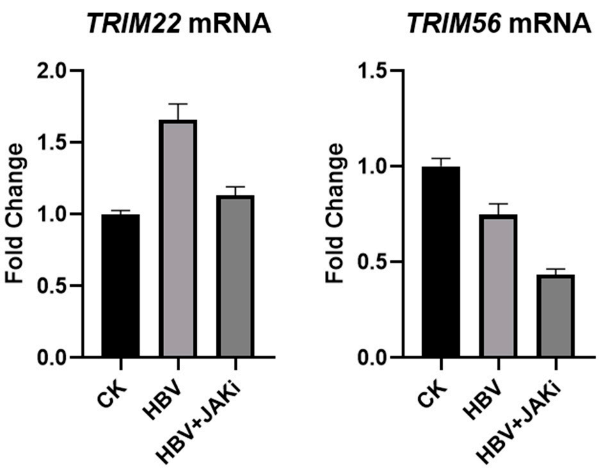

Supplemental Figure S2. *TRIM22* and *TRIM56* expression upon treatment with JAKi (10 µM) and HBV transfection for 48 h in HepG2.

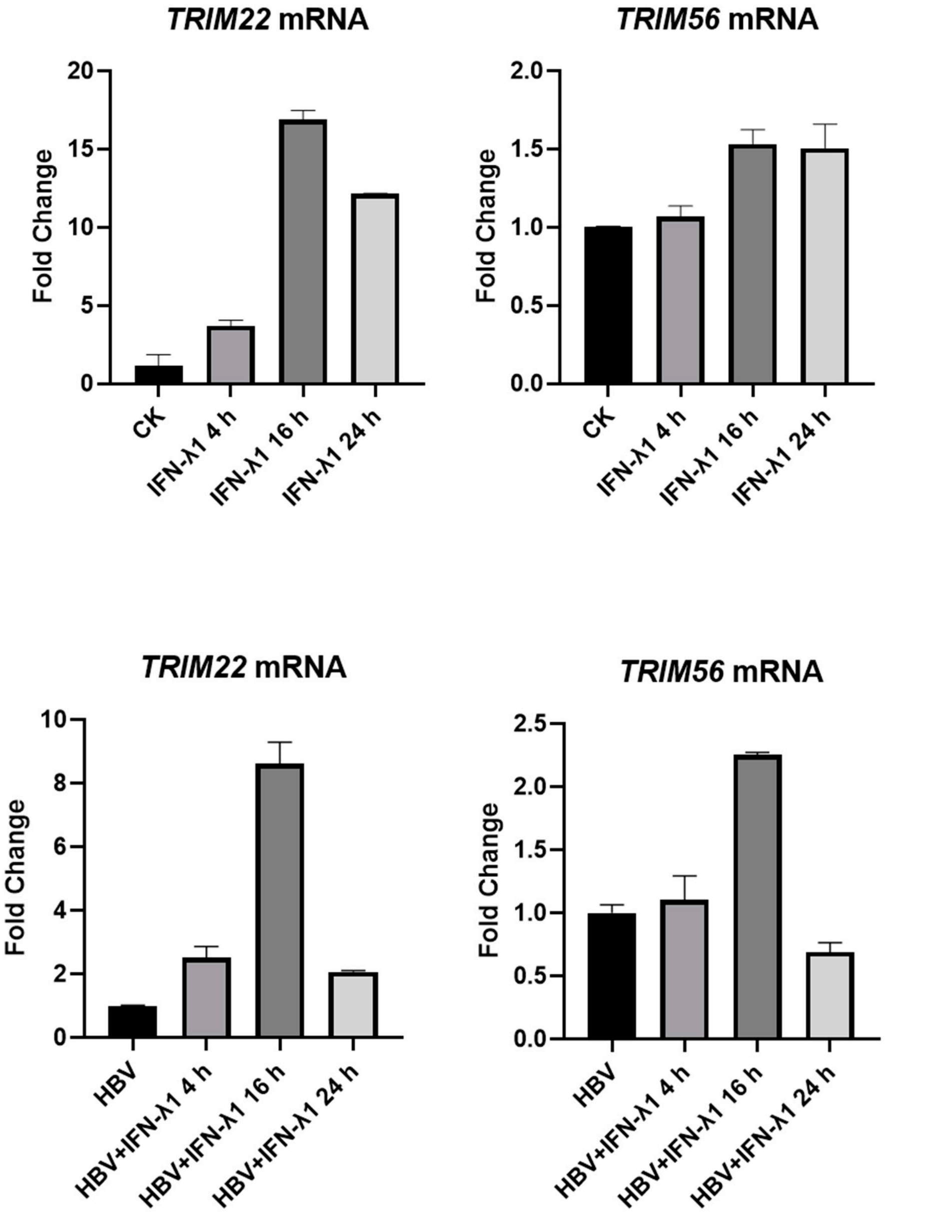

Supplemental Figure S3. IFN-λ1 treatment induces *TRIM22* and *TRIM56* expressions

in HEK293 cells with or without HBV transfection.

Supplemental Table S1. Primers or siRNA sequences.

Primers or siRNA	Sequence 5′-3′
RIG-I-Forward	AGTTGCTGATGAAGGCATTGAC
RIG-I-Reverse	GCACTTGCTACCTCTTGCTCTT
MAVS-Forward	GTCACTTCCTGCTGAGA
MAVS-Reverse	TGCTCTGAATTCTCTCCT
NF-κB-Forward	GAAATTCCTGATCCAGACAAAAAC
NF-κB-Reverse	ATCACTTCAATGGCCTCTGTGTAG
IRF3-Forward	ACCAGCCGTGGACCAAGAG
IRF3-Reverse	TACCAAGGCCCTGAGGCAC
IRF7-Forward	GAAGAGCCTGGTCCTGGTGAA
IRF7-Reverse	GGAAGCACTCGATGTCGTCAT
IRF9-Forward	GCTCTTCAGAACCGCCTACTT
IRF9-Reverse	GGCTCTCTTCCCAGAAATTCA
CXCL10-Forward	CTGATTTGCTGCCTTATCTTTCT
CXCL10- Reverse	ATGCAGGTACAGCGTACAGTTCT
CXCL11-Forward	GCCTTGGCTGTGATATTGTGTG
CXCL11- Reverse	TGCCACTTTCACTGCTTTTACC
MX1-Forward	TCCGACACGAGTTCCACAAAT
MX1-Reverse	AAAGCCTGGCAGCTCTCTACC
ISG15-Forward	CGCAGATCACCCAGAAGATCG
ISG15-Reverse	TTCGTCGCATTTGTCCACCA
TRIM22-Forward	AATGTGCTGGATAACCTGGCA
TRIM22-Reverse	TCTACTGACGATCCCCTCAAC
TRIM56-Forward	GCCTGCATACCTACTGCCAAG
TRIM56-Reverse	GCAGCCCATTGACGAAGAAGT
IFIT1-Forward	AGAAGCAGGCAATCACAGAAAA
IFIT1-Reverse	CTGAAACCGACCATAGTGGAAAT

SP110-Forward	CCTATGCCATACACAAGCCATT
SP110-Reverse	CCTCTCCAGTTGGGTGAGAAT
HBV pgRNA-Forward	TGTTCAAGCCTCCAAGCT
HBV pgRNA-Reverse	GGAAAGAAGTCAGAAGGCAA
HBV mRNA-Forward	GCACTTCGCTTCACCTCTGC
HBV mRNA-Reverse	CTCAAGGTCGGTCGTTGACA
HBV DNA-Forward	GTG TCT GCG GCG TTT TAT CA
HBV DNA-Reverse	GAC AAA CGG GCA ACA TAC CTT
HBx-Forward	GTCTCCTCTGACTTCAACAGCG
HBx-Reverse	ACCACCCTGTTGCTGTAGCCAA
HBV P-Forward	GGAGTGTGGATTCGCACTCC
HBV P-Reverse	TCGGGAAAGAATCCCAGAGGATTGG
GAPDH-Forward	ACAACTTTGGTATCGTGGAAGG
GAPDH-Reverse	GCCATCACGCCACAGTTTC
siHBx-sense	UGUGCACUUCGCUUCACCU
siHBx-antisense	AGGTGAAGCGAAGTGCACA
si*TRIM22*-sense	CAAUAUGGCUACUGGGUUA
si*TRIM22*-antisense	UAACCCAGUAGCCAUAUUG
si*TRIM56*-sense	GAGCAGCGACUUCCUGGCCUGUAAA
si*TRIM56*-antisense	UUUACAGGCCAGGAAGUCGCUGCUC
siNC-sense	UUCUCCGAACGUGUCACGUTT
siNC-antisense	ACGUGACACGUUCGGAGAATT
